# Association of daily copayments with use of hospital care among medicare advantage enrollees

**DOI:** 10.1186/s12913-019-4770-1

**Published:** 2019-12-12

**Authors:** John P. McHugh, Laura Keohane, Regina Grebla, Yoojin Lee, Amal N. Trivedi

**Affiliations:** 1 Columbia University, Mailman School of Public Health, 722 West 168th Street, 4th Floor, New York, NY 10032 USA; 2 Department of Health Policy, Vanderbilt University School of Medicine, 2525 West End Avenue, Suite 1200, Nashville, TN 37203 USA; 3 Center for Gerontology and Health care Research, Brown University, 121 South Main Street, Providence, RI 02903 USA; 4 Department of Health Services Policy and Practice, Brown University, 121 South Main Street, Providence, RI 02903 USA; 5 Providence Veterans Affairs Medical Center, 830 Chalkstone Avenue, Providence, RI 02908 USA

**Keywords:** Medicare advantage, Hospital utilization, Cost-sharing, Managed care

## Abstract

**Background:**

While the traditional Medicare program imposes a deductible for hospital admissions, many Medicare Advantage plans have instituted per-diem copayments for hospital care. Little evidence exists about the effects of changes in cost-sharing for hospital care among the elderly. Changing inpatient benefits from a deductible to a per diem may benefit enrollees with shorter lengths of stay, but adversely affect the out-of-pocket burden for hospitalized enrollees with longer lengths of stay.

**Methods:**

We used a quasi-experimental difference-in-differences study to compare longitudinal changes in proportion hospitalized, inpatient admissions and days per 100 enrollees, and hospital length of stay between enrollees in MA plans that changed inpatient benefit from deductible at admission to per diem, intervention plans, and enrollees in matched control plans – similar plans that maintained inpatient deductibles. The study population included 423,634 unique beneficiaries enrolled in 23 intervention plans and 36 matched control plans in the 2007–2010 period.

**Results:**

The imposition of per-diem copayments were associated with adjusted declines of 1.3 admissions/100 enrollees (95% CI − 1.8 to − 0.9), 6.9 inpatient days/100 enrollees (95% CI − 10.1 to − 3.8) and 0.7 percentage points in the probability of hospital admission (95% CI − 1.0 to − 0.4), with no significant change in adjusted length of stay in intervention plans relative to control plans. For persons with 2 or more hospitalizations in the year prior to the cost-sharing change, adjusted declines were 3.5 admissions/100 (95% CI − 8.4 to 1.4), 31.1 days/100 (95% CI − 75.2 to 13.0) and 2.2 percentage points in the probability of hospitalization (95% CI − 3.8 to − 0.6) in intervention plans relative to control plans.

**Conclusions:**

Instituting per-diem copayments was associated with reductions in number of admissions and hospital stays, but not length of stay once admitted. Effects of inpatient cost-sharing changes were magnified for persons with greater baseline use of hospital care.

## Background

Cost sharing is a common technique utilized by health insurers to “share” a portion of an enrollee’s health expenditures with the enrollee. This often takes the form of a payment at the point of service (co-payment) or payment for a fixed percentage of the cost of a given health service (co-insurance). In the hospital setting, this could also be a lump sum payment at admission (a deductible), or a payment for each day in the hospital (a per diem) [1, 2].

The Medicare program has used cost sharing in various forms since its inception in 1965. Medicare enrollees are responsible for 20% coinsurance for physician visits and large inpatient deductibles for hospital admissions, with no cap on out-of-pocket spending. The role of cost sharing and its clinical and economic effects in the Medicare program are topics of ongoing health policy debate. A study of the commercially insured found substantial increases in hospital cost sharing from 2009 to 2013 [3].

There is relatively little evidence to guide policymakers about the impact of greater cost sharing in the Medicare program. The landmark RAND Health Insurance Experiment, a randomized trial of cost sharing in health care, found that persons with higher coinsurance rates used less care and had lower spending than those with more generous insurance [4]. However, the RAND experiment ended in 1982 and excluded the elderly, limiting its generalizability to contemporary Medicare beneficiaries. The imposition of an inpatient deductible in the United Mine Workers Health Plan in 1977 was associated with a 45% decline in the probability of having a hospitalization [5]. Recent studies demonstrate that in response to increased outpatient copayments, Medicare beneficiaries reduced their use of outpatient services, but made greater use of hospital care [6, 7]. To our knowledge, studies of the Medicare population related to incentives and cost sharing in the hospital have been limited to the Medigap program which provides supplemental insurance to cover inpatient deductibles and other copayments. These studies also find increased inpatient utilization for those experiencing decreased cost sharing through supplemental Medigap insurance [8–11]. There are important differences between Medicare Advantage and Medigap. Most Medigap policies eliminate inpatient cost sharing altogether, which is a much larger change than our study and presumably less relevant for Medicare Advantage, purchasing Medigap reduces or eliminates cost sharing for many other services besides inpatient care, and the effect of cost sharing may differ in Medicare Advantage given supply-side, managed care constraints that are not found in traditional Medicare. Recent studies related to Medicare and Medicare Advantage focused on prescription drug use and adherence [12, 13], skilled nursing facility utilization [14], or Medicare Advantage enrollment [15]. The lack of inpatient utilization studies in the broader Medicare fee-for-service and Medicare Advantage programs is an important gap since hospital costs are the largest component of Medicare spending and the Medicare Part A deductible is the largest single out-of-pocket expense in the traditional Medicare benefit structure, $1340 in 2018 [16]. Further, as there has been increasing policy interest in raising cost sharing in the traditional Medicare program to control spending, one strategy has been to reduce the generosity of supplemental plans and expose enrollees to first-dollar cost sharing, the findings in this study would help to inform the debate [17].

Medicare Advantage plans, which currently enroll 31% of all beneficiaries, have experimented with changes to inpatient cost sharing [18]. A common change has been to eliminate the deductible and impose a daily copayment for each day of hospital care [19]. In 2010, “virtually all Medicare Advantage plans, 94%, required enrollees to share in the costs of inpatient care. 81% percent imposed copayments, 2% imposed coinsurance, and 11% used both. Among Medicare Advantage plans charging copayments for inpatient care, 79% charged a copayment per day, 16% charged a copayment per stay, and 5% charged both copayments per stay and per day.” [20] A deductible is typically exceeded during the first day of a hospital stay, leaving no financial incentive for a patient to leave the hospital earlier. In contrast, a per diem structure retains an incentive for a patient to leave the hospital throughout his or her stay. Thus, changing a plan’s benefit structure from a deductible to a per diem could mean lower out-of-pocket spending for beneficiaries with shorter lengths of stay, but greater out-of-pocket costs for hospitalized beneficiaries with longer lengths of stay, and subsequently could lead to decreased utilization. This study highlights the tradeoffs of this benefit change (potentially lower inpatient utilization but perhaps much higher cost-sharing requirements for sicker enrollees). In this study, we examined the impact of per-diem copayment and increased levels of cost sharing on the use of hospital care among Medicare Advantage enrollees age 65 and older. We hypothesized that changing the inpatient benefit structure from a deductible at admission to a per diem will result in reduced hospital utilization at the plan level.

## Methods

### Data source and study population

We obtained individual-level data from the Medicare Healthcare Effectiveness Data and Information Set (HEDIS) maintained by the Centers for Medicare and Medicaid Services (CMS) for the years 2007 through 2010. HEDIS contain individual-level data on Medicare Advantage (MA) enrollees’ use of hospital care. Individuals were matched to the Medicare beneficiary summary file to determine their demographic characteristics. Monthly information on health plan benefits for all Medicare plans was used to identify each plan’s cost-sharing requirement for inpatient hospitalizations. Information on health plan characteristics is publicly available on the CMS website.

We identified 33 plans that changed their inpatient benefit from a deductible at admission to a per diem (daily copayment), hereafter referred to as intervention plans. The intervention plans were identified across any two-year timeframe between 2007 and 2010 (e.g., 2007–2008, 2008–2009, or 2009–2010), with the intervention plans changing from a deductible in the first year of the two-year period to a per diem copayment in the second year of the two-year period. We found 223 plans that had no change in any inpatient or post-acute cost sharing across any one of the two-year timeframes between 2007 and 2010, hereafter referred to as control plans. Because changes in outpatient cost sharing can have an effect on hospital use and skilled nursing facility (SNF) or ambulatory care may substitute for hospital use, we limited the intervention and control plans to those that did not change, or made minimal change, to physician office or SNF cost sharing. Additionally, to mitigate any issues with co-insurance, we limited the intervention and control plans to those that did not impose co-insurance. In other words, the intervention plans imposed inpatient deductibles in year one and per diem copayments in year 2 whereas control plans imposed inpatient deductibles only in both years 1 and 2.

From the 33 intervention plans and 223 control plans, we utilized 1:n matching to match on the basis of contract year, tax status (i.e., for-profit or not-for-profit), geography, and deductible amount. We required plans to match based on contract year and tax status. Then, matching was prioritized by state, contract, neighboring state, division, region, and baseline inpatient deductible. Of the 33 case plans, 28 were matched to control plans. We excluded 5 pairs with incomplete data across the two analysis years or pairs with low volume (less than 150 admissions) in either of the analysis years. Our final sample consisted of 23 intervention plans matched to 36 control plans.

From our initial sample of 565,075 unique individuals, we limited our sample to those beneficiaries 65 years of age and older, excluding 99,303 individuals (17.8%), and who were not dually enrolled in Medicaid, excluding another 42,138 individuals (7.5%), resulting in our main analytic sample of 423,634 unique individuals enrolled in the intervention and control plans during our observation period.

### Variables

The main outcome variables were inpatient utilization as measured by inpatient admissions per 100 enrollees, inpatient days per 100 enrollees, proportion hospitalized and the mean length of stay. Length of stay was calculated as the total number of inpatient days divided by the total number of inpatient admissions.

The primary independent variables were an indicator variable for whether the health plan changed from an inpatient deductible to a per diem (1 for intervention and 0 for controls), an indicator variable for time (0 for the year before the intervention plans changed the inpatient benefit and 1 for the year after), and an interaction term between those variables.

We determined whether each individual received a Part D subsidy, which can serve as a proxy covariate for low income. Since we do not have individual level income, the Part D subsidy can serve as a valid substitute since Part D low-income subsidy recipients have limited assets and a maximum income of 150% of the federal poverty level [21]. Those receiving part D subsidies were subject to inpatient and outpatient copayments, since we excluded dual eligible enrollees.

Covariates included age category (65 to 74 years or older than 74 years), sex, race or ethnic group (black, white, other), and low-income Part D subsidy. To account for differences in plan benefits, we added the copayment amount for primary care and specialist office visits and the monthly premium amount. To account for any temporal trends in inpatient utilization, we also included a fixed effect for the calendar year.

### Analyses

We used a difference-in-difference approach to assess the effect of plans changing from an inpatient deductible to a per diem benefit. This method accounts for time-invariant trends in outcomes by subtracting the change in inpatient utilization in control plans from the concurrent change in intervention plans that changed the inpatient cost-sharing benefit (hereafter referred to as difference-in-differences estimates) [22, 23].

We fitted one-part generalized linear models that included independent variables and covariates described above. We specified a negative binomial distribution and identity link for inpatient admissions and days per 100 enrollees and inpatient length of stay, and a binomial distribution for the proportion hospitalized. We ran each model using PROC GENMOD and clustered standard errors at the plan level to account for correlation among enrollees.

We conducted a sensitivity analysis that restricted the population to those who were continuously enrolled in the same plans for a full 24 months, the 12 months before and after the benefit change. These enrollees exhibited a much greater increase in utilization, perhaps indicating a sicker population with a higher likelihood of hospitalization in the second year. To account for exit and entrance of enrollees from health plans, we conducted an additional sensitivity analysis that considered all enrollees irrespective of the number of months of enrollment. Higher baseline utilization among these enrollees could be due to the inclusion of decedents who will often have high concentrations of hospital use at the end of life. Because there may be selection issues in the disenrollment from a plan, enrollment into a plan or the decision to stay in a plan based on the plan benefits, we also assessed the characteristics of enrollees that exited their plan, those that entered a plan after the intervention plans changed their benefit structures and those that remained with their plan as well as disenrollment rates from intervention and control plans.

To evaluate whether pre-policy trends in inpatient utilization were similar in intervention and control plans, we estimated difference-in-difference effects comparing annual changes in all outcomes during the two-year time period prior to the change in inpatient benefits. In other words, for an intervention plan that changed from a deductible in 2008 to a per diem in 2009, we analyzed the plan’s differences in inpatient utilization between 2007 and 2008. None of the estimates reached conventional levels of statistical significance at the 95% level. (Appendix Table 4) We also conducted a falsification test utilizing dual eligible enrollees that were excluded from our primary analysis since they are not subject to cost sharing. None of the estimates reached conventional levels of statistical significance at the 95% level. (Appendix Table 5).

All analyses were performed with the use of SAS software, version 9.4. Results are reported with two-tailed *P*-values or 95% confidence intervals. Brown University’s Human Research Protections Office and the CMS Privacy Board approved the study protocol.

## Results

**Fig. 1:**
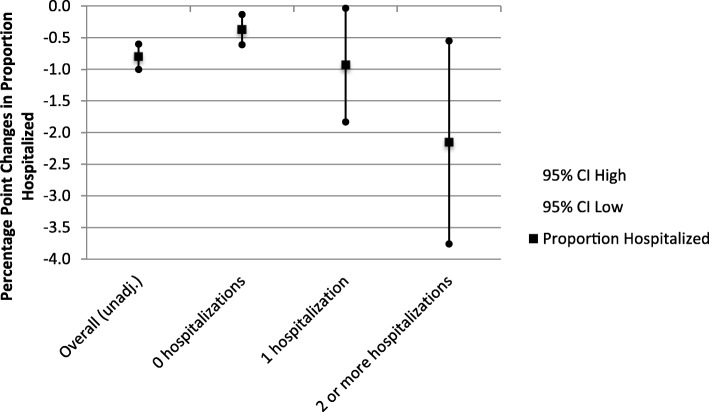
Difference-in-difference Estimates in the Proportion of Patients with a Hospital Admission, by the Number of Hospitalizations in the Year Before the Cost-sharing Change. Proportion Hospitalized (with 95% Confidence Intervals)

In the year before intervention plans replaced the inpatient deductible with a per-diem copayment, the average inpatient deductible in intervention plans was $376 (interquartile range [IQR], $250 to $500) and in control plans was $349 (IQR, $200 to $600). In the year after intervention plans changed their inpatient benefit, intervention plans replaced their inpatient deductible with a $165 (IQR, $110 to $225) average daily copayment and the average inpatient deductible in control plans remained unchanged, by design. (Table 1) 71.6% of all hospitalized enrollees had only 1 inpatient admission in a given year, 18.6% had 2 inpatient admissions and the remaining 9.8% had 3 or more inpatient admissions in any given year. (Hospitalization data not reported in Table 1, but used to generate Fig. 1) The demographic characteristics of enrollees (e.g., age, sex, race, etc.) in the intervention and control plans were similar. (Table 1) Skilled nursing facility cost sharing was unchanged in the intervention and control plans. For outpatient cost sharing, intervention plans exhibited a $4 increase in average specialist copayments and a $2 increase in average primary care copayments compared to a $1 decrease in average primary care copayments and no change in specialist copayments in the control plans. Emergency department copayments remained unchanged in the intervention and control plans. (Table 1) Average monthly premiums for intervention plans decreased slightly while control plan premiums remained stable. Intervention plans had a higher percentage of zero premium plans as compared to control plans. (Table 1).

**Table 1:** Enrollee and Benefit Characteristics in Intervention and Control Plans

Variable	Intervention Plans		Control Plans		All Plans
	Year before deductible to per diem change	Year after deductible to per diem change	Year before intervention plans changed benefit	Year after intervention plans changed benefit	Yearly Average 2007 to 2010
*Enrollee Characteristics*					
Enrollees	182,452	216,549	267,774	291,546	4,261,061
Hospitalized Enrollees	26,731	30,356	38,353	43,350	664,112
Age – yr	75.3 (6.9)	75.0 (7.0)	75.6 (6.8)	75.6 (7.0)	74.4 (7.0)
Female sex – %	53.8	53.5	53.5	53.1	51.9
Race – %					
White	89.3	89.3	90.4	90.4	84.7
Black	5.8	5.8	4.0	4.0	8.8
Other	5.0	5.0	5.6	5.6	6.6
Part D subsidy – %	4.1	4.2	4.6	4.5	5.4
*Benefit Characteristics*					
Number of Plans	23	23	42	42	1975
Average star rating	2.9	2.6	2.8	2.9	2.9
Average # of Enrollees	7933	9415	6376	6942	2157
Plan Type					
HMO	91.3	91.3	69.0	69.0	53.0
PPO	8.7	8.7	26.2	26.2	22.4
PFFS	0.0	0.0	2.4	2.4	17.0
Other	0.0	0.0	2.4	2.4	7.6
Mean deductible – $	$376		$349	$349	$412
Mean per diem (days 1–5) – $		$165			$161
Mean PCP copayment – $	$11	$13	$13	$12	$14
Mean Spec. copayment – $	$21	$25	$21	$21	$25
Mean emergency department copayment – $	$50	$50	$49	$49	$49
Zero premium plans – %	65.2	60.9	45.2	45.2	58.5
Mean plan premium amount A/B – $	$20	$18	$31	$31	$24
SNF requires prior hospital stay – % of plans	0.0	0.0	14.3	14.3	10.1
SNF benefits include cost sharing for first 20 days of SNF stay – % of plans					
Coinsurance	0.0	0.0	0.0	0.0	14.7
Deductible	0.0	0.0	0.0	0.0	1.2
Copayment	100.0	100.0	92.9	92.9	45.0

Unadjusted inpatient admissions per 100 enrollees decreased in intervention plans by 0.4 admissions per 100 enrollees from the year before the benefit change to the year after the benefit change. In control plans, inpatient admissions per 100 enrollees increased by 1.1 admissions per 100 enrollees. Therefore, the difference-in-difference estimate of intervention plans relative to control plans was − 1.4 admissions per 100 enrollees (95% CI, − 1.8 to − 0.9). Adjusting for age, sex, race and Part D subsidy and including a year fixed effect resulted in a difference-in-difference estimate of − 1.3 admissions per 100 enrollees (95% CI, − 1.8 to − 0.9) for intervention plans relative to controls. (Table 2).

**Table 2:** Use of Hospital Care in Intervention Plans that Replaced a Hospital Deductible with a Per-diem Copayment Compared to Concurrent Trends in Matched Control Plans that Maintained a Hospital Deductible

	Intervention Plans		Control Plans		Difference- in-Difference Estimates	
	Year Before Benefit Change	Year After Benefit Change	Year Before Intervention Plan Changes	Year After Intervention Plan Changes	Unadjusted	Adjusted
Beneficiaries enrolled for full 12 months in either year						
Inpatient admissions per 100 enrollees	20.0	19.6	19.7	20.8	−1.4*** (−1.8 to −0.9)	−0.9** (− 1.4 to − 0.3)
Inpatient days per 100 enrollees	87.0	86.9	81.9	88.2	−6.3*** (−9.3 to −3.3)	−4.2* (−7.9 to − 0.5)
Proportion hospitalized (%)	13.9	13.5	13.9	14.4	−1.0*** (− 1.3 to − 0.7)	− 0.5** (− 0.9 to − 0.1)
Mean inpatient length of stay	4.0	4.1	4.0	4.0	0.0 (− 0.1 to 0.1)	0.0 (− 0.1 to 0.2)
Beneficiaries enrolled for full 24 months in same plan						
Inpatient admissions per 100 enrollees	24.4	29.2	24.0	29.7	−0.9** (−1.4 to − 0.4)	−0.6 (− 1.2 to 0.0)
Inpatient days per 100 enrollees	122.8	154.4	117.8	152.0	−2.6 (−6.0 to 0.8)	−2.0 (− 6.3 to 2.4)
Proportion hospitalized (%)	10.9	13.3	10.8	13.9	−0.6** (− 0.9 to − 0.3)	−0.3* (− 0.7 to 0.0)
Average inpatient length of stay	4.5	5.0	4.5	4.9	0.1 (−0.0 to 0.2)	0.0 (−0.1 to 0.1)
Beneficiaries enrolled for any amount of time						
Inpatient admissions per 100 enrollees	28.5	28.2	28.8	30.0	−1.5*** (−2.0 to − 1.0)	−0.9** (− 1.5 to − 0.4)
Inpatient days per 100 enrollees	153.2	154.2	150.7	158.3	−6.6*** (− 10.0 to −3.2)	−3.6 (−7.7 to 0.6)
Proportion hospitalized (%)	12.2	11.8	12.5	13.2	−1.1*** (− 1.4 to −0.8)	−0.5** (− 0.9 to − 0.2)
Average inpatient length of stay	4.9	5.0	4.9	4.9	0.0 (−0.1 to 0.1)	0.0 (0.0 to 0.1)

Note: All values are unadjusted, except those in the final column

*** *p* < .0001, ** *p* < .01, * *p* < .05

We observed similar results for inpatient days per 100 enrollees and for the proportion of enrollees hospitalized. Unadjusted inpatient days per 100 enrollees in intervention plans decreased by − 6.3 days per 100 enrollees relative to control plans (95% CI, − 9.3 to − 3.3) and the unadjusted proportion of enrollees hospitalized in intervention plans decreased by 1.0 percentage points relative to control plans (95% CI, − 1.3 to − 0.7). The adjusted difference-in-difference estimate of inpatient days per 100 enrollees in intervention plans relative to control plans was − 6.9 days (95% CI, − 10.1 to − 3.8). The adjusted difference-in-difference estimate of the proportion of enrollees hospitalized in intervention plans relative to control plans was − 0.7 percentage points (95% CI, − 1.0 to − 0.4). Neither unadjusted nor adjusted difference-in-difference estimates of mean length of stay reached conventional measures of significance at the 95% level. Results were similar when considering enrollees continuously enrolled for an entire 24-month period in the same plan and when examining beneficiaries enrolled for any amount of time. (Table 2).

We observed similar enrollee characteristics and inpatient utilization for beneficiaries staying enrolled in the same plan across intervention and control plans in the year prior to the benefit changes. We also see similar demographic characteristics among enrollees that exited plans in the year prior to exiting the plan and among enrollees that entered plans in the year prior to entering one of our study plans. A higher proportion of enrollees that exited intervention and control plans were hospitalized in the baseline year, 24%, as compared to those enrollees remaining in the same plan, 11%. There was no difference, though, when comparing intervention and control plans. Inpatient utilization for enrollees entering intervention and control plans in the year prior to entering was also similar. Beneficiaries disenrolled at a slightly higher rate from intervention plans, 16%, as compared to control plans, 15.5%. (Table 3).

**Table 3:** Enrollee Characteristics and Utilization in Intervention and Control Plans for Beneficiaries Staying, Exiting and Entering Plans

	Disenrollment Rate – %	Age – yr (sd)	Female sex – %	White – %	Black – %	Other race – %	Part D subsidy – %	Inpatient Admissions per 100 enrollees	Inpatient days per 100 enrollees	Proportion hospitalized (%)	Mean inpatient length of stay
Beneficiaries staying enrolled in same plan (utilization in period before benefit change in intervention plans)											
Intervention Plans	n/a	75.3 (6.9)	53.6	89.1	6.0	4.9	4.2	24.4	122.8	10.9	4.5
Control Plans	n/a	75.1 (6.8)	52.4	91.2	4.0	4.8	4.0	24.0	117.8	10.8	4.5
Beneficiaries EXITING a plan (utilization in period before benefit change in intervention plans)											
Intervention Plans	16.0	76.0 (7.6)	51.8	86.9	7.0	6.1	4.9	40.2	222.5	24.0	5.3
Control Plans	15.5	77.4 (7.5)	52.9	90.1	4.0	5.9	4.9	41.2	227.5	24.0	5.3
Beneficiaries ENTERING a plan in year after benefit change in intervention plans (utilization in period before entering plan)											
Intervention Plans	n/a	72.2 (6.9)	52.0	88.1	6.3	5.6	4.7	15.3	59.8	10.8	3.7
Control Plans	n/a	73.1 (7.5)	51.0	89.6	5.5	4.8	4.2	14.5	59.1	10.3	3.9

For enrollees who were not hospitalized in their baseline year, the unadjusted difference-in-difference estimate of the proportion of enrollees hospitalized in intervention plans relative to control plans was − 0.4 percentage points (95% CI, − 0.6 to − 0.1). For those enrollees hospitalized 1 time in their baseline year, the unadjusted difference-in-difference estimate was − 0.9 percentage points (95% CI, − 1.8 to − 0.03). Finally, for enrollees hospitalized 2 or more times in the baseline year, the unadjusted difference-in-difference estimate of the proportion of enrollees hospitalized in intervention plans relative to control plans was − 2.2 percentage points (95% CI, − 3.8 to − 0.6). (Fig. 1) Unadjusted difference-in-difference estimates of admissions and days per 100 enrollees and average length of stay across strata of baseline hospitalizations did not reach conventional measures of significance at the 95% level.

## Discussion

We studied the effect of changing an inpatient insurance benefit from a deductible at admission to a per diem (charge per day) in a large sample of Medicare Advantage enrollees age 65 and older. We found that the change in benefit structure was associated with significant declines in inpatient admissions and days per 100 enrollees. We also found a 0.7 adjusted percentage point reduction in the proportion of enrollees hospitalized in intervention plans relative to control plans. Enrollees with greater use of hospital care in the year prior to the cost-sharing change experienced greater declines in hospital utilization, perhaps indicating that patients, once hospitalized, were more acutely aware of the cost-sharing burden and took steps to avoid future admissions, or, perhaps, the plan itself more actively managed the patient to avoid additional inpatient expenses. Once enrollees were hospitalized, though, we did not find any significant difference in the adjusted length of stay between intervention and control plans.

Our findings are consistent with research on inpatient utilization and its association with supplemental Medigap insurance. Medigap coverage, or decreased cost sharing, has been found to be associated with increases in inpatient utilization, and we find a directionally similar result, with *increased* cost sharing associated with *decreases* in inpatient utilization, though it should be noted that the increased cost sharing only applies, on average, to patients hospitalized 3 days or more (70.7% of hospitalized patients in our sample). Our findings are also broadly consistent with two studies of hospital cost sharing among non-elderly populations. The United Mine Workers Study demonstrated that the imposition of a $250 hospital deductible in 1977 led to a “45% decline in the probability of a hospital admission” from a baseline of 6.8%, but resulted in an increase in length of stay among those hospitalized [5]. Our results show about a 7% relative reduction in the probability of a hospitalization from a baseline of about 20%. The RAND experiment also found increases in cost sharing were associated with reductions in the likelihood of seeking care, but not the intensity of care once the patient was hospitalized [2, 4]. We also observe a reduction in the proportion hospitalized and reduction in inpatient admissions and days per 100 among enrollees exposed to the cost-sharing change. However, like the RAND study, we did not observe a corresponding increase in length of stay, perhaps because this outcome is more strongly influenced by the decisions of hospital physicians with relatively little impact by patients [24]. A central but often unappreciated finding from the RAND study is that cost sharing was not associated with reductions in the use of health services *after* patients initiate contact with the health care system.

Inpatient utilization remains the most expensive component of Medicare spending [25]. Therefore, if the purpose of the change in benefit structure was to reduce overall spending on hospital care, the intervention plans in our study likely achieved this objective by reducing aggregate inpatient utilization. However, there were substantial increases in out-of-pocket spending among hospitalized patients, particularly among patients with longer lengths of stay. For instance, a person with a median length of stay would experience expected out-of-pocket costs in the baseline year of $376. In the year after the benefit change, the out-of-pocket costs for an enrollee in an intervention plan with a median length of stay of 4.4 days would increase by 93% to $726. However, persons with longer lengths of stays would have substantially greater increases in out-of-pocket costs. For instance persons at the 75th percentile of length of stay (5.5 days) could expect to pay $908 dollars for an admission after the cost-sharing changes went into effect, an increase of 141%. This highlights the importance for Medicare Advantage enrollees, and for those helping consumers navigate the market, to understand the full package of benefits as it is quite possible some enrollees hospitalized after the benefit change were unaware of the higher copayment until after the hospitalization.

Strengths of our study include the use of a large sample of over 400,000 beneficiaries in 59 MA plans across the country. By observing the entire structure of each plans’ benefit, we were able to identify plans that only changed their inpatient cost sharing, without making significant changes to skilled nursing facility, physician office or emergency department cost sharing. We matched plans by geographic region and confirmed that hospitalization utilization trends in intervention and control plans were similar prior to the change in cost sharing. To our knowledge, this is the first study to quantify the impact of changes in inpatient cost sharing among Medicare Advantage enrollees. We do, though, find greater cost sharing is associated with reduced inpatient utilization, similar to the studies done in the Medigap program.

Our study has limitations. First, we are unable to observe MA plans’ strategies that, apart from changes in benefits, may have impacted hospital utilization. For instance, intervention plans may have implemented more stringent utilization management practices, or made changes in their network of preferred hospitals. However, this would assume that these strategies were implemented in intervention plans and not in control plans and occurred at the same time that inpatient cost sharing was changed. Second, hospitalization and length of stay decisions are complex and include many factors and decision makers, including physicians, care managers, other providers, and patients; our study did not directly observe these processes and exclusively relied on administrative data to quantify changes in inpatient utilization. Third, since traditional Medicare applies an inpatient deductible with no per diem, it would be difficult to generalize the findings to traditional Medicare beneficiaries. However, current policy debates related to cost sharing in the traditional Medicare program could benefit from these findings and, more broadly, the findings may also have implications for benefit design for other kinds of insurance, both public and private. Fourth, it is possible that enrollees selectively disenrolled from intervention plans, anticipating hospitalizations in the following year. However, we ran our regressions on enrollees that exited plans in the year they were included in our study and in the year after exiting one of our study plans and found no significant difference in utilization in our adjusted difference-in-difference models and in all but one (proportion hospitalized) of our unadjusted models. Fifth, we are limited in the covariates we have access to with our data, so there may have been substantial unobserved differences in key variables such as comorbidity and clinical complexity among enrollees in intervention and control plans that could have influenced our findings. However, we did not observe large baseline differences in hospital utilization between intervention and control plans, nor did we observe differences in utilization among those enrollees entering or exiting the intervention and control plans. Finally, we are limited to a small set of plans that met our matching criteria. There are differences between our study population and the overall Medicare Advantage population, therefore there are limits to the generalizability of our study. However, given the paucity of studies on this topic, this provides a foundation for future studies to better understand the effects of inpatient cost sharing on utilization.

## Conclusion

In conclusion, we found that changing from a deductible to a per diem copayment structure was associated with reductions in utilization of inpatient care among Medicare Advantage enrollees, particularly among those with greater use of hospital care prior to the copayment change. These reductions appear to be driven by decreases in the number of admissions and the probability of hospitalization, without significant changes in length of stay. Although the use of hospital care, but not the duration of admissions, may be sensitive to daily out-of-pocket costs, the financial burden of changing from a deductible to a per-diem falls heavily on seniors with longer hospital stays.

## Data Availability

The data that support the findings of this study are available from the Research Data Assistance Center (ResDAC) but restrictions apply to the availability of these data, which were used under license for the current study, and so are not publicly available.

## Appendix 1

**Table 4:** Use of Hospital Care in Case Plans that Replaced a Hospital Deductible with a Per-diem Copayment Compared to Concurrent Trends in Matched Control Plans that Maintained a Hospital Deductible, in the Time Period Prior to the Benefit Change

	Intervention Plans		Control Plans		Difference- in-Difference Estimates	
	2 Years Before Benefit Change	1 Year Before Benefit Change	2 Years Before Intervention Plan Changes	1 Year Before Intervention Plan Changes	Unadjusted	Adjusted
All continuously enrolled beneficiaries						
Inpatient admissions per 100 enrollees	20.0	19.0	20.1	19.6	−0.4 (−1.3 to 0.4)	−0.4 (− 1.2 to 0.4)
Inpatient days per 100 enrollees	86.2	82.3	84.3	85.2	−4.8 (−9.7 to 0.1)	−3.1 (−8.0 to 1.8)
Proportion hospitalized (%)	14.3	14.2	13.8	13.7	−0.1 (− 0.6 to 0.4)	−0.5 (−1.0 to 0.0)
Average inpatient length of stay	4.1	4.1	4.1	4.1	−0.1 (− 0.2 to 0.1)	−0.1 (− 0.2 to 0.1)

## Appendix 2

**Table 5:** Use of Hospital Care in Intervention Plans that Replaced a Hospital Deductible with a Per-diem Copayment Compared to Concurrent Trends in Matched Control Plans that Maintained a Hospital Deductible, Dual Eligible Enrollees Only

	Intervention Plans		Control Plans		Difference- in-Difference Estimates	
	Year Before Benefit Change	Year After Benefit Change	Year Before Intervention Plan Changes	Year After Intervention Plan Changes	Unadjusted	Adjusted
Inpatient admissions per 100 enrollees	38.9	41.0	35.6	38.2	−0.5 (−2.9 to 2.0)	1.2 (−1.4 to 3.8)
Inpatient days per 100 enrollees	197.7	224.0	181.4	197.9	9.8 (−7.4 to 27.0)	19.4 (−0.7 to 39.6)
Proportion hospitalized (%)	22.8	22.6	22.0	22.8	−1.1 (−2.2 to 0.1)	−0.3 (− 1.5 to 1.0)
Average inpatient length of stay	4.6	4.8	4.8	4.9	0.2 (−0.1 to 0.5)	0.2 (−0.1 to 0.7)

## Abbreviations

CI: Confidence Interval
CMS: Centers for Medicare and Medicaid Services
HEDIS: Healthcare Effectiveness Data and Information Set
HMO: Health Maintenance Organization
IQR: Interquartile Range
MA: Medicare Advantage
PCP: Primary Care Physician
PFFS: Private Fee-For-Service
PPO: Preferred Provider Organization
SNF: Skilled Nursing Facility
